# Biological Variation in Biochemistry Analytes in Laboratory Guinea Pigs (*Cavia porcellus*)

**DOI:** 10.3390/vetsci10100621

**Published:** 2023-10-17

**Authors:** Gabriele Rossi, Kwei-Farn Liu, Helen Kershaw, Dayna Riddell, Timothy H. Hyndman, Deborah Monks, Gabrielle C. Musk

**Affiliations:** 1 School of Veterinary Medicine, Murdoch University, Murdoch, WA 6150, Australia; kwei-farn.liu@murdoch.edu.au (K.-F.L.); t.hyndman@murdoch.edu.au (T.H.H.); 2 Centre for Animal Production and Health, Murdoch University, Murdoch, WA 6150, Australia; 3 Animal Care Services, University of Western Australia, Crawley, WA 6009, Australia; helen.kershaw@uwa.edu.au (H.K.); dayna.riddell@aff.gov.au (D.R.); gabrielle.musk@uwa.edu.au (G.C.M.); 4 Brisbane Bird and Exotics Veterinary Service, Greenslopes, QLD 4120, Australia; deborah.monks@bbevs.com.au

**Keywords:** index of individuality, reference change value, reference intervals, within-individual variability, between-individual variability

## Abstract

**Simple Summary:**

This is the first report of biological variation data in guinea pigs for biochemical analytes (measurands) routinely measured during health screening. Information on biological variation provides an insight into whether the observed value for the biochemical analyte is significant or not for that individual. This information is also used to determine whether population-based reference intervals are appropriate to aid in interpreting blood biochemistry results. Biological variation is defined as the within-subject and between-subject random fluctuation around a homeostatic point. In this study, we have calculated the index of individuality, which helps to understand whether subject-based or population-based reference intervals are more appropriate, in order to better identify significant changes in the same subject over time (e.g., during the follow-up period). Our results show that population-based reference intervals should be used on their own only for creatinine and potassium. Individual reference intervals should be used only for glucose. All other markers will require an interpretation that considers both subject-based and population-based reference intervals. It is important to note that most of the biochemical results in the healthy guinea pigs enrolled in our study had biochemical results outside the published reference intervals; thus, information on biological variation helps clinicians and researchers with the interpretation of biochemical profiles.

**Abstract:**

Biological variation (BV) describes the physiological random fluctuation around a homeostatic set point, which is a characteristic of all blood measurands (analytes). That variation may impact the clinical relevance of the changes that are observed in the serial results for an individual. Biological variation is represented mathematically by the coefficient of variation (CV) and occurs within each individual (CV_I_) and between individuals in a population (CV_G_). Biological variation data can be used to assess whether population-based reference or subject-based reference intervals should be used for the interpretation of laboratory results through the calculation of the index of individuality (IoI). This study aimed to determine the biological variations, calculate the IoI and reference change values (RCV) of clinical chemistry analytes in an outbred strain colony of Hartley guinea pigs (GPs), and set the quality specifications for clinical chemistry analytes. Blood was collected from 16 healthy adult laboratory colony GPs via jugular venipuncture at weekly intervals over six weeks. All the samples were frozen and analyzed in a single run. Analytical, CV_I_, and CV_G_ biological variations, together with the IoI and RCV, were calculated for each measurand. Based on the estimated BV, the calculated IoI was low for glucose, so individual reference intervals (RCV) should be used. The majority of the measurands should be interpreted using both population-based and subject-based reference intervals as the IoIs were intermediate.

## 1. Introduction

Guinea pigs are a valuable and validated experimental animal model in research and toxicology studies [1,2,3]. In vivo studies are often characterized by sequential sample collections to compare pre- and post-treatment effects or to monitor the effects of drug administration over time [1]. Even in companion animals, the emergence of the “preventative care” philosophy means that veterinarians have more access to, and are more commonly evaluating, serial data from individual patients [4].

When interpreting the results of biochemical analyte measurements, the values for an individual patient are commonly compared to reference intervals (RI) that are determined from a healthy population of guinea pigs (population-based reference intervals). However, every analytical measurement has three inherent sources of variation: pre-analytical, analytical, and biological variation (BV) [5]. Pre-analytical variation is defined as the variation in sample analyte concentrations caused by factors that occur before sample analysis: animal handling, sample collection, sample processing, and sample storage. Analytical variation is defined as the variation attributable to the laboratory instrument.

Biological variation describes the physiological random fluctuation around a homeostatic point, which is a characteristic of all blood measurands [5,6,7]. That variation may impact the clinical relevance of changes in serial results for an individual [8]. Biological variation is represented mathematically by the coefficient of variation (CV) and occurs within each individual (CV_I_) and between individuals in a population (CV_G_). 

Analytes with marked inter-individual variation have broad population-based RIs. For these analytes, clinically relevant changes measured in an individual over time can still fall within the population-based RI, especially if the intra-individual variation is relatively small. For such analytes, clinicians should use subject-based reference intervals to monitor those clinically important changes. Biological variation data can therefore be used to assess whether population-based reference or subject-based reference intervals should be used for the interpretation of laboratory results. This is achieved through the calculation of the index of individuality (IoI), which expresses the relationship between the CV_I_ and CV_G_ [6,7,8,9]. Measurands with high IoI (>1.67) have a higher intra-individual variation and relatively lower inter-individual variation, suggesting that they would be best evaluated by population-based reference intervals [6,7,9]. In contrast, analytes with low IoI (<0.7) are appropriately interpreted with the reference change value (RCV). The RCV is used to assess whether the percentage difference between a homeostatic set point and measured result is clinically relevant and can facilitate an understanding of whether the observed changes in serial patient data are likely to be clinically relevant [6,7,8,9]. Those analytes with intermediate individuality (0.7 ≤  IoI  ≤  1.7) should be assessed with a consideration of both population-based and subject-based RI, depending on the clinical picture of the patient [6,7,9].

Consequently, understanding the biologic variation of biochemical analytes allows clinicians to determine the best method of evaluating serial changes in any particular analyte.

Population-based reference intervals have been calculated for some measurands in inbred strain 13/N [10], Dunkin Hartley [11,12], and Weiser-Maples [13] guinea pigs. Biological variation data are available for many species and measurands either in scientific publications [14,15,16,17,18,19] or databases available on the vetbiologicalvariation.org website (accessed on 27 September 2023) [20]. 

No biological variation data have been reported in guinea pigs despite the increasing popularity of guinea pigs as pocket pets and their more frequent presentation to veterinary clinics. Thus, the aims of this study were: (1) to determine the biological variations; (2) to calculate the IoI and RCV of clinical chemistry analytes in an outbred strain colony of Hartley guinea pigs; and (3) to set the quality specifications for clinical chemistry analytes.

## 2. Materials and Methods

### 2.1. Study Population

This prospective project was conducted on an outbred strain colony of non-genetically modified Hartley tri-color guinea pigs bred by the University of Western Australia (UWA). The study was approved by the UWA Animal Ethics Committee (approval number: 2020/ET000166). The guinea pigs were accommodated in a PC2 facility accredited by the Association for Assessment and Accreditation of Laboratory Animal Care. The guinea pigs were housed in single-sex pens measuring 175 cm × 55 cm (9625 cm^2^ floor area) × 35 cm height. The bedding material was coarse aspen chips (ABEDD SIA, Kalnciems, Latvia) and pine shavings (Snoozle Plus; WA Pine Shavings, Bellevue, WA, Australia). The lighting intensity ranged from 150 to 950 lux on a schedule of 12 h of white light, 2 h of red, and 10 h off, which was automated via the programmable lighting system Controlsoft. The room temperature was maintained between 18 °C and 24 °C, and the humidity was the ambient building humidity of 30–70%. All guinea pigs were provided with *ad lib* access to a commercially supplied diet (High Energy Guinea Pig diet; Specialty Feeds, Glen Forrest, WA, Australia) that was steam sterilized. In addition, but only on weekdays, autoclaved oaten hay (Feedman Stockfeeds, Martin, WA, Australia) and fresh vegetables (kale, bok choy, broccoli, red capsicum, and occasionally herbs) were provided. The guinea pigs were provided with *ad lib* access to tap water supplemented by sodium ascorbate (vitamin C) (Vitamin C (Ascorbic Acid); Animal Health Solutions, Belmont, WA, Australia) at 1 g/L, which was replaced daily (seven days a week).

All guinea pigs were adults with the first sample collected when they were 5 months old. Both sexes were enrolled in the study.

The guinea pigs were weighed and monitored weekly until the commencement of blood collection, which involved assessing their activity, demeanor, coat condition, appetite, and water intake. After the procedures began, the animals were monitored twice weekly with the addition of observing the venipuncture site until the end of the experiment. More frequent monitoring was performed if there were any abnormalities observed of any of the aforementioned parameters. Prior to the start of the blood sampling, the guinea pigs were gently handled and restrained to habituate them to the position required for the collection of blood from a jugular vein. This handling was performed for a few minutes up to three times per week for two weeks.

### 2.2. Sampling

Blood was collected from the jugular vein once a week for six weeks. The collection time was standardized as much as possible, starting around 9 am and finishing around 11 a.m. every day. The fur around the jugular veins was clipped, and a liberal layer of a topical local anesthetic cream (EMLA (eutectic mix of local anesthetic) 5% cream; 25 mg/g lignocaine, 25 mg/g prilocaine; Aspen Pharmacare Australia, St Leonards, NSW, Australia) was applied. One hour was given for the local anesthetic to take effect before venipuncture was performed. The guinea pigs were restrained in lateral recumbency against the holder’s body, with the neck extended. Gentle digital pressure was then applied in the jugular groove to engorge the jugular vein, which could not be visualized. The blood samples collected from the jugular vein were transferred into plain blood tubes; a limit of four attempts was allowed to limit tissue trauma and distress to the animals. Between 0.5 mL and 2 mL of whole blood was collected each week. The animals were observed for 15 min following venipuncture to monitor for hemorrhage or bruising. Guinea pigs with any abnormalities (such as bruising around the venipuncture site) were monitored daily until the issue was resolved.

The blood samples were left to stand to allow clot formation for approximately one hour before they were centrifuged at 753 rcf for 10 min. The serum was then separated and stored frozen at −20 °C for nine to 24 weeks before being sent in dry ice to Murdoch University for analysis. 

At the end of the study, the guinea pigs were anaesthetized with isoflurane in a 10 L chamber (4% isoflurane (Isothesia; 99.9% isoflurane; Covetrus, Portland, ME, USA)) in 100% oxygen at 4 L/min. When the animals were deeply anaesthetized, they were transferred to a breathing system with a facemask for the delivery of 1–3% isoflurane in 100% oxygen at 2 L/min. The depth of anesthesia was determined by the lack of a toe-pinch reflex. The guinea pigs were then positioned in dorsal recumbency with the forelimbs extended cranially. The location of the heart was determined by the palpation over the ribs of the area with the strongest heartbeat. A sterile needle was inserted between the ribs in line with the palpated heartbeat, and blood was drawn. Following blood withdrawal, with the needle still in place, the guinea pigs were euthanized using 1 mL of 325 mg/mL sodium pentobarbitone (Lethabarb; Virbac, Milperra, NSW, Australia). The cardiac blood collected was used for the calculation of the analytical variation of the measurands.

### 2.3. Analytical Methods

The biochemical analytes included in the profile are summarized in Table 1; electrolytes (Cl, K, Na) were included in the profile only if the volume of serum was greater than 250 μL.

A minimum of 150 μL was required to perform the analyses; in the situation where sample volumes were less than this, the sample was excluded from the study (Table 2).

All analyses were performed with the Cobas Integra 400 Plus (Roche Diagnostics, North Ryde, NSW, Australia) chemistry analyzer. Calibration (calibrator for automated systems c.f.a.s., Roche Diagnostics) and two levels of quality control (PreciControl ClinChem Multi 1 and 2, PCCC1 and PCCC2, Roche Diagnostics) were performed before the analysis, and the acceptability of performance was guided by Westgard Multirules, as per ASVCP guidelines [21].

All the serum samples (jugular and cardiac) were thawed and tested by one operator in the same analytical run to minimize analytical variation. The remaining volume in all the cardiac samples was then analyzed in duplicate to permit the estimation of the coefficient of analytical variation (CV_A_).

### 2.4. Statistical Analysis

Data analysis was performed following the recommendations for biological studies [9]. Statistical analyses were conducted using Stata (v18.0, StataCorp, College Station, TX, USA). 

The outliers were identified by scrutinizing the box and whisker plots of the observed data, and by inspecting the residual plots and normal quantile-quantile plots of the fitted models. 

A linear mixed-effects model was fitted to the data for each measurand using restricted maximum likelihood (ReML; Table 1). Each guinea pig had a random intercept. Two correlation structures were compared for the within guinea pig variation: independent and auto-regressive lag-1 (AR1). Likelihood ratio (LR) tests were used to compare the two correlation structures. If the *p*-value was less than 0.05, the AR1 correlation structure was chosen (i.e., consecutive measurements are more closely correlated to each other than non-consecutive measurements). If the *p*-value of the LR test was at least 0.05, the independent correlation structure was chosen (i.e., repeated measurements are assumed to be independent of one another). No covariates were included in the models. Residual plots and normal quantile-quantile (Q-Q) plots were used to assess heteroskedasticity (Supplementary Figure S1) and the normality of the measurands (Supplementary Figure S2), respectively.

A fixed-effects model was used instead of a mixed-effects model for creatinine and potassium because the within guinea pig variation far exceeded the between guinea pig variation. This was formally tested using an LR test. This meant that for these two measurands, there was no difference between the mixed-effects model (that specified repeated measures were from the same guinea pig) and the fixed-effects model that assumed that all measurements from all guinea pigs were independent of one another.

For the mixed-effects models, the CV_G_ was calculated as the standard deviation of the normal distribution of the random intercepts (the between guinea pig variation) divided by the predicted value of the measurand. The CV_I_ was the standard deviation of the residual variation of the model (the within guinea pig variation) divided by the predicted value of the measurand. For both the CV_G_ and CV_I_, the 95% confidence intervals were calculated using the method described by Vangel [22]. There is no separation between the within guinea pig variation and between guinea pig variation with the fixed-effects models, as all measurements are treated as being independent of one another. This means that the CV_G_ and CV_I_ cannot be calculated from fixed-effects models. 

In order to estimate the CV_A_, the biochemical analytes of the cardiac samples were measured in duplicate.

The index of individuality (IoI), reference change value (RCV), and quality specifications for imprecision, inaccuracy, and total errors were then derived from the indices of the BV (Table 3) [23].

## 3. Results

This biological variation study population used 16 guinea pigs, of which 8 were females and 8 were males. One male was housed alone for 4 weeks due to fighting with cage mates. One male had bruising at the venipuncture site but was retained in the study. A total of 76 jugular samples were obtained, 19 of which were less than 1 mL. Due to the small sample volume, 9 samples had their biochemistry assessed only, and 55 samples had results from both biochemistry and electrolytes (Table 2). A total of 26 cardiac samples were analyzed in duplicate on the same day for an estimation of the CV_A_.

Desirable analytical imprecision (CV_A_:CV_I_ ≤ 0.5) was observed for all the measurands except sodium, which confirms that the sample size in this study had suitable power for the BV data calculation of most of the biomarkers [9].

**Figure 1:**
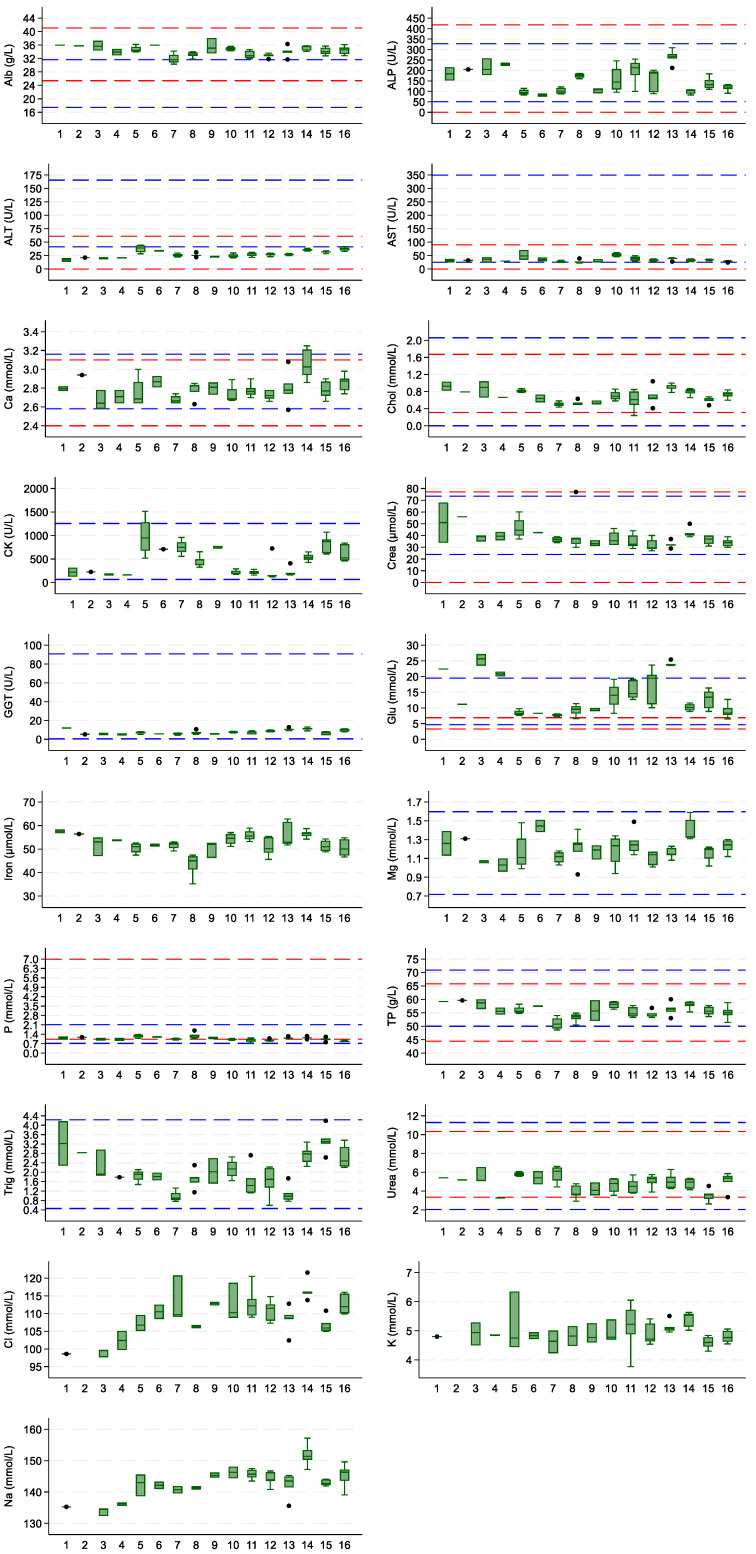
Boxplots for each of the 16 guinea pigs for each measurand. Boxes indicate the I-III interquartile range (IQR); the horizontal lines indicate the median values (quartile II); upper whiskers extend to the highest observed value within 1.5 × IQR of quartile III; and lower whiskers extend to the lowest observed value within 1.5 × IQR of quartile I. Black dots indicate outliers (values exceeding 1.5 × IQR from quartile I or III). The dotted lines represent the population-based reference intervals for guinea pigs published by Baldrey [24] (in red) and Rabe [25] (in blue).

A total of five outliers were removed: one for sodium due to an analytical error, the other for AST due to hemolysis, and three others (two for CK and one for AST) that could not be explained, but clearly were not part of their measurands’ normal distribution. All other data were retained and included in the calculation of the BV. The individual data for all measurands are shown in Figure 1. Context is provided through the inclusion of population-based reference intervals from two studies [24,25]. Individual data, without these reference intervals, are shown in Supplementary Figure S3.

The values of CV_I_, CV_G_, and CV_A_ are summarized in Table 4, along with the derived IoI, RCV, and quality specifications calculated using two different methods.

A unidirectional Z-score of 1.65 was applied when calculating the RCVs for ALP, ALT, AST, and GGT. The rest of the biomarkers required two-sided interpretation, and hence, a Z-score of 1.96.

It was not possible to calculate the CV_I_ and CV_G_ for creatinine and potassium because they could not be modeled with the mixed-effects model; thus, the resultant IoI, RCV, and analytical performance goals were also not calculated. 

Only glucose showed low individuality (IoI < 0.7), which suggests that the use of a subject-specific RCV is appropriate for this test.

## 4. Discussion

The standard laboratory guinea pig for research is the Dunkin Hartley, an outbred, smooth-coated, albino strain. This strain was first developed by Dunkin and Hartley in 1926 and is commercially available from several laboratory breeders [26]. The Dunkin Hartley is the most common guinea pig strain used in biomedical research, particularly for studies of asthma [27,28,29], allergy [30,31], infectious disease [2,32,33], reproduction [34,35,36], and osteoarthritis [37,38]. Minimally invasive blood tests, such as complete blood counts and serum biochemistry profiles, are often collected for diagnostics and laboratory analyses. Hematology and serum chemistry population-based reference intervals are available for Dunkin Hartley [11,12], but to the authors’ knowledge, there is no published information about the biological variation of biochemistry markers in guinea pigs. Once the biological variability is known, the index of individuality can be calculated, which can be used to inform clinicians and researchers on the best approach for data interpretation. Data from biological variations can also be used to calculate the RCV. This study demonstrated that the RCV is suitable only for glucose due to a low index of individuality. Creatinine and potassium measurements were shown to be independent of one another (i.e., not specific to individual guinea pigs), and the population-based RIs are appropriate for these two measurands.

Most measurands in the present study had intermediate indices of individuality and the use of both population-based reference intervals and RCV may be helpful in diagnosis and for the monitoring of changes in serial results. In the case of serial measurements, it is appropriate to analyze the RCV between two consecutive measurements rather than to compare results to population-based RI [6,39]. Given that in laboratory animals sequential blood collections are commonly performed, the RCV may be a practical tool to detect small but clinically significant changes when the results are still within the population-based reference intervals.

In veterinary medicine, recommendations are available to inform researchers how to correctly perform a biological variation study [9]. The minimum number of study subjects is dependent on the CV_A_:CV_I_ ratio. When the CV_A_:CV_I_ ratio is ≤ 0.5, a minimum number of 10–15 study subjects are needed and specimens should be obtained weekly over at least 4–6 weeks. For all the biochemical analytes measured in the present study (with the exception of sodium), the CV_A_:CV_I_ ratio was lower than 0.5. Due to the unsuccessful blood collection from some animals or the limited sample volume, a smaller sample size than recommended was available for all measurands, and this is a limitation of the study.

Although duplicate measurements are recommended, due to the limited blood sample volume that was collected from the guinea pigs, the BV was calculated based on a single measurement, which represents a limitation of this study with an effect on the statistical power. However, the analytical precision of each measurand was assessed in duplicate over a total of 26 different samples. The samples used to calculate the analytical variation were collected by cardiocentesis as a terminal procedure under general anesthesia. The analytical precision was high for all measurands, with a CV_A_ that was often <1.0%. Therefore, the contribution of analytical variation on the overall variability was minimal. In rodents and reptiles, it is known that there are differences in hematologic and serum biochemical parameters based on the venipuncture site [40,41,42]. To avoid potential bias, only the samples collected by cardiocentesis were used for the calculation of analytical variability, and only the samples collected by jugular venipuncture were used for the calculation of intra- and inter-individual variability.

Given that the guinea pigs enrolled in this study were housed in a laboratory facility, the environmental conditions, diet, and age were standardized. These conditions helped to reduce the pre-analytical variability which can be present when the BV is calculated in pet animals. A limitation of this study is the difference in the storage time of the samples collected in successive weeks because the samples were batch-tested on the same day to limit analytical variation. Previous studies suggested that most of the biochemical analytes were stable in human plasma and serum after 30 days of storage at −20 °C, and in rat serum after 90 days of storage at −20 °C [43,44]. However, statistically significant changes in several biochemical analytes have been noted in canine serum and heparinized plasma samples from 90- to 240-day storage at −70 °C. Among those analytes, the changes in the concentrations of GGT, LDH, and magnesium were potentially clinically relevant [45]. Therefore, the prolonged and variable storage time of these guinea pig sera at −20 °C, varying from nine to 24 weeks, potentially increased pre-analytical variability.

The first blood samples were collected as soon as the guinea pigs reached adulthood (5 months) to avoid the age-related changes reported in previously published studies [11]. As examples, ALP and phosphorus significantly decreased with age, likely due to the decline in bone growth as animals reached skeletal maturity. In the guinea pig, bone growth is purported to cease by 4 months of age. For the same reason, elderly guinea pigs were not included in the study due to variations in chemical measurements such as BUN, creatinine, and calcium, which increase in elderly animals due to reduced renal function [11].

The index of individuality can inform clinicians if subject-based or population-based reference intervals should be used for biochemical data interpretation. Population-based reference intervals for different strains of guinea pigs have been published [10,11,12]; however, the RCV may be more beneficial, particularly for the interpretation of sequential measurements. 

The RCV showed a broad range: as low as 5.9% for sodium and as high as 133.8% for CK. The RCV reflects the clinical interpretation of biochemical analytes. For example, smaller (absolute) changes in electrolytes are clinically significant, while bigger (absolute) changes should be observed with liver or muscle enzymes to be clinically relevant. The use of RCV helps clinicians and researchers to make an objective judgment on the relevance of the changes observed and can be used either on laboratory or pet guinea pigs. 

Further studies to investigate the effect of gender either within or between sexes on the biochemical values or the degree of biological variation are required, and an investigation of the storage stability of frozen serum samples should also be conducted. Due to the limited sample volume, it was not possible to include those aims in the present study.

## 5. Conclusions

In this study, we evaluated, for the first time, the biological variation of biochemical measurands in a population of laboratory guinea pigs. The results revealed that the majority of these measurands were characterized by an intermediate individuality and that the interpretation of test results should be performed considering both individual- and population-based RIs. When sequential blood collections are performed, the RCV can also be an extremely useful tool to identify clinically significant changes.

## Figures and Tables

**Table 1:** Methods used for biomarker measurements and the linear range. The model used for each measurand is also listed. For mixed-effects (ME) models, the correlation structure is also listed. FE = fixed-effects model. AR1 = auto-regressive lag-1. IND = independent.

Measurand	Method	Measuring Range	Model (ME or FE)	Correlation Structure (AR1 or IND)
**Albumin (Alb)**	Bromocresol green	2–60 g/L	ME	IND
**Alkaline phosphatase (ALP)**	Colorimetric IFCC	3–1200 U/L	ME	IND
**Alanine transaminase (ALT)**	Colorimetric IFCC w/o P5P	2–700 U/L	ME	AR1
**Aspartate aminotransferase (AST)**	Colorimetric IFCC w/o P5P	2–700 U/L	ME	IND
**Calcium (Ca)**	Colorimetric NM-BAPTA	0.2–5.0 mmol/L	ME	IND
**Cholesterol (Chol)**	Colorimetric	0.1–20.7 mmol/L	ME	IND
**Creatine kinase (CK)**	UV-test	7–2000 U/L	ME	IND
**Creatinine (Crea)**	Jaffé	18–1300 μmol/L	FE	N/A
**Gamma-glutamyl transferase (GGT)**	Enzymatic colorimetric IFCC	3–1200 U/L	ME	AR1
**Glucose (Glu)**	Enzymatic with hexokinase	0.24–40 mmol/L	ME	IND
**Iron**	FerroZine	0.9–179 μmol/L	ME	AR1
**Magnesium (Mg)**	Colorimetric Chlorophosphonazo III	0.15–2.5 mmol/L	ME	IND
**Phosphorus (P)**	Endpoint method with sample blanking	0.1–6.46 mmol/L	ME	IND
**Total protein (TP)**	Colorimetric biuret	2–120 g/L	ME	IND
**Triglycerides (Trig)**	Enzymatic colorimetric	0.1–10 mmol/L	ME	IND
**Urea**	Kinetic test with urease and glutamate dehydrogenase	0.5–40 mmol/L	ME	IND
**Electrolytes (Sodium, Na; Chloride, Cl; Potassium, K)**	Ion-selective electrodes, using undiluted samples (ISE)		K: FE Na, Cl: ME	K: N/A Na, Cl: IND

**Table 2:** A record of the tests performed using the samples from each week. Cells shaded green denote that both biochemistry and electrolytes were measured. Yellow indicates that only biochemistry was performed as there was not enough serum for electrolyte analysis. A white cell marked with an “X” means the sample was not successfully collected or was of insufficient volume to perform any tests. ID = guinea pig identifier. F = female. M = male. W = week of study.

ID Sex	1 F	2 F	3 F	4 F	5 M	6 M	7 M	8 M	9 M	10 F	11 F	12 F	13 F	14 M	15 M	16 M
**W1**	X	X		X		X			X			X				
**W2**		X	X	X	X	X	X	X	X							
**W3**	X	X			X				X	X			X			
**W4**		X		X		X	X									
**W5**	X		X			X				X						
**W6**	X	X	X	X												

**Table 3:** A summary of the formulae used in this study.

	Formulae
**Index of individuality**	$\mathrm{IoI}=\frac{\sqrt{{\mathrm{CV}}_{I}^{2}+{\mathrm{CV}}_{A}^{2}}}{{\mathrm{CV}}_{G}}$
**Reference change value**	$\mathrm{RCV}=Z\times \sqrt{2}\times \sqrt{{\mathrm{CV}}_{I}^{2}+{\mathrm{CV}}_{A}^{2}}$ where Z = 1.65 for a 95% confidence interval if only increasing or decreasing concentration is of clinical concerns (e.g., enzymes); Z = 1.95 for a 95% confidence interval if both high and low concentrations are clinically important.
**CV Opt**	Recommended optimal analytical variation (imprecision) based on CV_A_ < 0.25CV_I_
**CV Des**	Recommended desirable analytical variation (imprecision) based on CV_A_ < 0.5CV_I_
**CV Min**	Recommended minimally acceptable analytical variation (imprecision) based on CV_A_ < 0.75CV_I_
**Bias Opt**	Recommended optimal bias (inaccuracy) based on <0.125(${\mathrm{CV}}_{I}^{2}$ + ${\mathrm{CV}}_{G}^{2}$)^1/2^
**Bias Des**	Recommended desirable bias (inaccuracy) based on <0.25(${\mathrm{CV}}_{I}^{2}$ + ${\mathrm{CV}}_{G}^{2}$)^1/2^
**Bias Min**	Recommended minimally acceptable bias (inaccuracy) based on <0.375(${\mathrm{CV}}_{I}^{2}$ + ${\mathrm{CV}}_{G}^{2}$)^1/2^
**TE_a_ Opt**	Recommended optimal allowable total error based on <1.65(0.25CV_I_) + 0.125(${\mathrm{CV}}_{I}^{2}$ + ${\mathrm{CV}}_{G}^{2}$)^1/2^
**TE_a_ Des**	Recommended desirable allowable total error based on <1.65(0.5CV_I_) + 0.25(${\mathrm{CV}}_{I}^{2}$ + ${\mathrm{CV}}_{G}^{2}$)^1/2^
**TE_a_ Min**	Recommended minimally acceptable allowable total error based on <1.65(0.75CV_I_) + 0.375(${\mathrm{CV}}_{I}^{2}$ + ${\mathrm{CV}}_{G}^{2}$)^1/2^
**Alt TE_a_ Opt**	Alternative recommended optimal allowable total error based on <1.65(CV Opt)
**Alt TE_a_ Des**	Alternative recommended desirable allowable total error based on <1.65(CV Des)
**Alt TE_a_ Min**	Alternative recommended minimally acceptable allowable total error based on <1.65(CV Min)

**Table 4:** Biological variation data and the analytical performance goals. For the within- (CV_I_) and between- (CV_G_) animal coefficients of variation, 95% confidence intervals are included. The units of measurement for the mean values are the same as Figure 1.

		Biological Variation						Traditional Quality Specifications Based on Biological Variation									Alternative TE_a_ Based on Biologic Variation		
	Mean	CV_I_ ± SD (%) (95% CI)	CV_G_ ± SD (%) (95% CI)	CV_A_ ± SD (%)	CV_A_:CV_I_	IoI	RCV (%)	CV Opt	CV Des	CV Min	Bias Opt	Bias Des	Bias Min	TEa Opt	TEa Des	TEa Min	Alt TE_a_ Opt	Alt TE_a_ Des	Alt TE_a_ Min
Alb	34.4	3.4 ± 1.2 (2.9–4.2)	3.0 ± 1.0 (2.2–3.6)	0.9 ± 1.6	0.3	1.2	9.84	0.86	1.71	2.57	0.57	1.14	1.71	2.0	4.0	6.0	1.4	2.8	4.2
ALP	156.8	22.3 ± 35.0 (18.9–27.4)	32.7 ± 51.3 (23.0–40.6)	0.4 ± 0.4	0.0	0.7	52.16	5.59	11.17	16.76	4.95	9.90	14.85	14.2	28.3	42.5	9.2	18.4	27.7
ALT	27.1	16.6 ± 4.5 (14.1–20.3)	20.4 ± 5.5 (14.6–24.9)	1.6 ± 1.2	0.1	0.8	38.93	4.15	8.30	12.45	3.29	6.58	9.86	10.1	20.3	30.4	6.8	13.7	20.5
AST	34.9	19.4 ± 6.8 (16.4–23.8)	19.2 ± 6.7 (13.7–23.6)	0.7 ± 1.2	0.0	1.0	45.30	4.85	9.70	14.55	3.41	6.82	10.24	11.4	22.8	34.2	8.0	16.0	24.0
Ca	2.8	3.9 ± 0.1 (3.3–4.7)	2.9 ± 0.1 (2.1–3.6)	0.5 ± 0.6	0.1	1.3	10.93	0.98	1.95	2.93	0.61	1.22	1.84	2.2	4.4	6.7	1.6	3.2	4.8
Chol	0.7	18.1 ± 0.1 (15.3–22.1)	16.2 ± 0.1 (11.6–19.8)	0.6 ± 0.7	0.0	1.1	50.16	4.52	9.04	13.56	3.04	6.07	9.11	10.5	21.0	31.5	7.5	14.9	22.4
CK	458.8	37.6 ± 172.5 (31.3–47.2)	56.8 ± 260.7 (37.6–74.6)	0.7 ± 1.1	0.0	0.7	104.24	9.40	18.80	28.20	8.51	17.03	25.54	24.0	48.0	72.1	15.5	31.0	46.5
GGT	7.6	22.1 ± 1.7 (18.7–27.1)	25.7 ± 1.9 (18.3–31.6)	3.2 ± 3.1	0.1	0.9	52.12	5.53	11.05	16.58	4.24	8.48	12.72	13.4	26.7	40.1	9.1	18.2	27.3
Glu	14.1	19.1 ± 2.7 (16.1–23.3)	41.8 ± 5.9 (28.9–52.7)	0.3 ± 0.2	0.0	0.5	52.81	4.76	9.53	14.29	5.74	11.48	17.23	13.6	27.2	40.8	7.9	15.7	23.6
Iron	52.4	7.1 ± 3.7 (6.0–8.6)	6.2 ± 3.3 (4.5–7.5)	0.7 ± 0.5	0.1	1.1	19.77	1.78	3.55	5.33	1.18	2.36	3.54	4.1	8.2	12.3	2.9	5.9	8.8
Mg	1.2	9.9 ± 0.1 (8.4–12.1)	6.9 ± 0.1 (5.0–8.4)	0.9 ± 0.6	0.1	1.4	27.56	2.48	4.95	7.43	1.51	3.01	4.52	5.6	11.2	16.8	4.1	8.2	12.3
P	1.1	10.5 ± 0.1 (8.9–12.7)	9.5 ± 0.1 (6.9–11.5)	0.6 ± 0.7	0.1	1.1	29.15	2.62	5.25	7.87	1.77	3.54	5.31	6.1	12.2	18.3	4.3	8.7	13.0
TP	56.0	3.5 ± 2.0 (3.0–4.2)	3.3 ± 1.8 (2.4–4.0)	0.5 ± 0.4	0.1	1.1	9.80	0.87	1.75	2.62	0.60	1.20	1.80	2.0	4.1	6.1	1.4	2.9	4.3
Trig	2.1	23.9 ± 0.5 (20.2–29.3)	30.9 ± 0.6 (21.9–38.3)	0.5 ± 0.4	0.0	0.8	66.24	5.97	11.95	17.92	4.89	9.77	14.66	14.7	29.5	44.2	9.9	19.7	29.6
Urea	4.8	15.4 ± 0.7 (13.0–18.7)	13.5 ± 0.6 (9.7–16.4)	1.3 ± 1.0	0.1	1.1	42.76	3.84	7.68	11.53	2.55	5.11	7.66	8.9	17.8	26.7	6.3	12.7	19.0
Cl	108.9	3.1 ± 3.4 (2.6–3.8)	4.4 ± 4.8 (3.1–5.5)	1.2 ± 1.0	0.4	0.8	9.24	0.78	1.56	2.34	0.68	1.36	2.03	2.0	3.9	5.9	1.3	2.6	3.9
Na	142.6	1.8 ± 2.6 (1.6–2.3)	3.0 ± 4.3 (2.1–3.7)	1.1 ± 1.2	0.6	0.7	5.86	0.45	0.90	1.35	0.44	0.87	1.31	1.2	2.4	3.5	0.7	1.5	2.2

## Data Availability

Not applicable.

## Supplementary Materials

The following supporting information can be downloaded at: https://www.mdpi.com/article/10.3390/vetsci10100621/s1, Figure S1: Residual plot of the model-fitted residual value to the model-fitted value. The units of measurement for the model-fitted values are as for Figure 1; Figure S2: Normal quantile-quantile (Q-Q) plot of the quantiles of the residuals from each linear mixed-effects model to the quantiles of a standard normal distribution. A linear relationship is consistent with the conclusion that the observed values for the measurand were normally distributed; Figure S3: Boxplot for each of 16 guinea pigs for each measurand without previously published reference intervals.

## References

[1] Birck M.M., Tveden-Nyborg P., Lindblad M.M., Lykkesfeldt J. (2014). Non-terminal blood sampling techniques in guinea pigs. J. Vis. Exp..

[2] Padilla-Carlin D.J., McMurray D.N., Hickey A.J. (2008). The guinea pig as a model of infectious diseases. Comp. Med..

[3] Struillou X., Boutigny H., Soueidan A., Layrolle P. (2010). Experimental animal models in periodontology: A review. Open Dent. J..

[4] Riggs S.M., Mitchell M.A., Tully T.N. (2009). GUINEA PIGS. Manual of Exotic Pet Practice.

[5] Fraser C.G. (2011). Reference change values. Clin. Chem. Lab. Med..

[6] Campora C., Freeman K.P., Baral R. (2018). Clinical application of biological variation data to facilitate interpretation of canine and feline laboratory results. J. Small Anim. Pract..

[7] Flatland B., Baral R.M., Freeman K.P. (2020). Current and emerging concepts in biological and analytical variation applied in clinical practice. J. Vet. Intern. Med..

[8] Walton R.M. (2012). Subject-based reference values: Biological variation, individuality, and reference change values. Vet. Clin. Pathol..

[9] Freeman K.P., Baral R.M., Dhand N.K., Nielsen S.S., Jensen A.L. (2017). Recommendations for designing and conducting veterinary clinical pathology biologic variation studies. Vet. Clin. Pathol..

[10] Genzer S.C., Huynh T., Coleman-Mccray J.D., Harmon J.R., Welch S.R., Spengler J.R. (2019). Hematology and Clinical Chemistry Reference Intervals for Inbred Strain 13/n Guinea Pigs (*Cavia porcellus*). J. Am. Assoc. Lab. Anim. Sci..

[11] Spittler A.P., Afzali M.F., Bork S.B., Burton L.H., Radakovich L.B., Seebart C.A., Moore A.R., Santangelo K.S. (2021). Age- and sex-associated differences in hematology and biochemistry parameters of Dunkin Hartley guinea pigs (Cavia porcellus). PLoS ONE.

[12] Waner T., Avidar Y., Peh H.C., Zass R., Bogin E. (1996). Hematology and clinical chemistry values of normal and euthymic hairless adult male Dunkin-Hartley guinea pigs (*Cavia porcellus*). Vet. Clin. Pathol..

[13] Kitagaki M., Yamaguchi M., Nakamura M., Sakurada K., Suwa T., Sasa H. (2005). Age-related changes in haematology and serum chemistry of Weiser-Maples guineapigs (*Cavia porcellus*). Lab. Anim..

[14] Smith S.M., Carney P.C., Prieto J.M., Miller M.L., Randolph J.F., Farace G., Peterson S., Bilbrough G., Peterson M.E. (2023). Biological variation of biochemical analytes determined at 8-week intervals for 1 year in clinically healthy cats. Vet. Clin. Pathol..

[15] Baral R.M., Freeman K.P., Flatland B. (2021). Analytical quality performance goals for symmetric dimethylarginine in cats. Vet. Clin. Pathol..

[16] Baral R.M., Dhand N.K., Freeman K.P., Krockenberger M.B., Govendir M. (2014). Biological variation and reference change values of feline plasma biochemistry analytes. J. Feline Med. Surg..

[17] Colburn M.E., Schnelle A.N., Wong Y.K., Whitmore E.M., Reilly J.D., Adamovicz L.A., Keller K.A., Allender M.C. (2022). SHORT-TERM BIOLOGICAL VARIABILITY OF HEMATOLOGY PARAMETERS IN THE BEARDED DRAGON (*POGONA VITTICEPS*). J. Zoo Wildl. Med..

[18] Perrin K.L., Kristensen A.T., Gray C., Nielsen S.S., Bertelsen M.F., Kjelgaard-Hansen M. (2020). BIOLOGICAL VARIATION OF HEMATOLOGY AND BIOCHEMISTRY PARAMETERS FOR THE ASIAN ELEPHANT (*ELEPHAS MAXIMUS*), AND APPLICABILITY OF POPULATION-DERIVED REFERENCE INTERVALS. J. Zoo Wildl. Med..

[19] Ruaux C.G., Carney P.C., Suchodolski J.S., Steiner J.M. (2012). Estimates of biological variation in routinely measured biochemical analytes in clinically healthy dogs. Vet. Clin. Pathol..

[20] VetBiologicalVariation. http://www.vetbiologicalvariation.org/.

[21] Flatland B., Freeman K.P., Friedrichs K.R., Vap L.M., Getzy K.M., Evans E.W., Harr K.E. (2010). ASVCP quality assurance guidelines: Control of general analytical factors in veterinary laboratories. Vet. Clin. Pathol..

[22] Vangel M.G. (1996). Confidence Intervals for a Normal Coefficient of Variation. Am. Stat..

[23] Flatland B., Camus M.S., Baral R.M. (2018). Analytical quality goals-a review. Vet. Clin. Pathol..

[24] Baldrey V., Ashpole I. (2012). Interpreting blood profiles in non-domestic small mammals. Vet. Times.

[25] Rabe H. (2011). Reference ranges for biochemical parameters in guinea pigs for the Vettest^®^8008 blood analyzer. Tierarztl. Prax. Ausg. K Kleintiere Heimtiere.

[26] Pritt S., Suckow M.A., Stevens K.A., Wilson R.P. (2012). Taxonomy and History. The Laboratory Rabbit, Guinea Pig, Hamster, and Other Rodents.

[27] Canning B.J., Chou Y. (2008). Using guinea pigs in studies relevant to asthma and COPD. Pulm. Pharmacol. Ther..

[28] Ricciardolo F.L., Nijkamp F., De Rose V., Folkerts G. (2008). The guinea pig as an animal model for asthma. Curr. Drug Targets.

[29] Adner M., Canning B.J., Meurs H., Ford W., Ramos Ramírez P., van den Berg M.P., Birrell M.A., Stoffels E., Lundblad L.K., Nilsson G.P. (2020). Back to the future: Re-establishing guinea pig in vivo asthma models. Clin. Sci..

[30] Aamir R., Safadi G.S., Mandelik J., Cornish K., Melton A.L., Pien L.C., Wagner W.O., Battisto J.R. (1996). A guinea pig model of hypersensitivity to allergenic fractions of natural rubber latex. Int. Arch. Allergy Immunol..

[31] Merayo-Lloves J., Calonge M., Foster C.S. (1995). Experimental model of allergic conjunctivitis to ragweed in guinea pig. Curr. Eye Res..

[32] Kumar M., Krause K.K., Azouz F., Nakano E., Nerurkar V.R. (2017). A guinea pig model of Zika virus infection. Virol. J..

[33] Hensel M.E., Arenas-Gamboa A.M. (2018). A Neglected Animal Model for a Neglected Disease: Guinea Pigs and the Search for an Improved Animal Model for Human Brucellosis. Front. Microbiol..

[34] Rocca M.S., Wehner N.G. (2009). The guinea pig as an animal model for developmental and reproductive toxicology studies. Birth Defects Res. B Dev. Reprod. Toxicol..

[35] Canizo J., Zhao C., Vandal K., Biondic S., Petropoulos S. (2022). O-234 The guinea pig embryo: A potential new model for human development. Hum. Reprod..

[36] Taylor D.K., Lee V.K., Suckow M.A., Stevens K.A., Wilson R.P. (2012). Chapter 25—Guinea Pigs as Experimental Models. The Laboratory Rabbit, Guinea Pig, Hamster, and Other Rodents.

[37] Musci R.V., Walsh M.A., Konopka A.R., Wolff C.A., Peelor F.F., Reiser R.F., Santangelo K.S., Hamilton K.L. (2020). The Dunkin Hartley Guinea Pig Is a Model of Primary Osteoarthritis That Also Exhibits Early Onset Myofiber Remodeling That Resembles Human Musculoskeletal Aging. Front. Physiol..

[38] Veronesi F., Salamanna F., Martini L., Fini M. (2022). Naturally Occurring Osteoarthritis Features and Treatments: Systematic Review on the Aged Guinea Pig Model. Int. J. Mol. Sci..

[39] Lund F., Hyltoft Petersen P., Fraser C.G. (2019). A dynamic reference change value model applied to ongoing assessment of the steady state of a biomarker using more than two serial results. Ann. Clin. Biochem..

[40] López-Olvera J.R., Montané J., Marco I., Martínez-Silvestre A., Soler J., Lavín S. (2003). Effect of venipuncture site on hematologic and serum biochemical parameters in marginated tortoise (*Testudo marginata*). J. Wildl. Dis..

[41] Fernández I., Peña A., Del Teso N., Pérez V., Rodríguez-Cuesta J. (2010). Clinical biochemistry parameters in C57BL/6J mice after blood collection from the submandibular vein and retroorbital plexus. J. Am. Assoc. Lab. Anim. Sci..

[42] Abatan O.I., Welch K.B., Nemzek J.A. (2008). Evaluation of saphenous venipuncture and modified tail-clip blood collection in mice. J. Am. Assoc. Lab. Anim. Sci..

[43] Kachhawa K., Kachhawa P., Varma M., Behera R., Agrawal D., Kumar S. (2017). Study of the Stability of Various Biochemical Analytes in Samples Stored at Different Predefined Storage Conditions at an Accredited Laboratory of India. J. Lab. Physicians.

[44] Cray C., Rodriguez M., Zaias J., Altman N.H. (2009). Effects of storage temperature and time on clinical biochemical parameters from rat serum. J. Am. Assoc. Lab. Anim. Sci..

[45] Thoresen S.I., Tverdal A., Havre G., Morberg H. (1995). Effects of storage time and freezing temperature on clinical chemical parameters from canine serum and heparinized plasma. Vet. Clin. Pathol..

