# Supplementary figures and images for: Biological Variation in Biochemistry Analytes in Laboratory Guinea Pigs (Cavia porcellus)
Source: Vet Sci. 2023 Oct 17;10(10):621. doi: 10.3390/vetsci10100621 (PMC10610888; doi:10.3390/vetsci10100621)

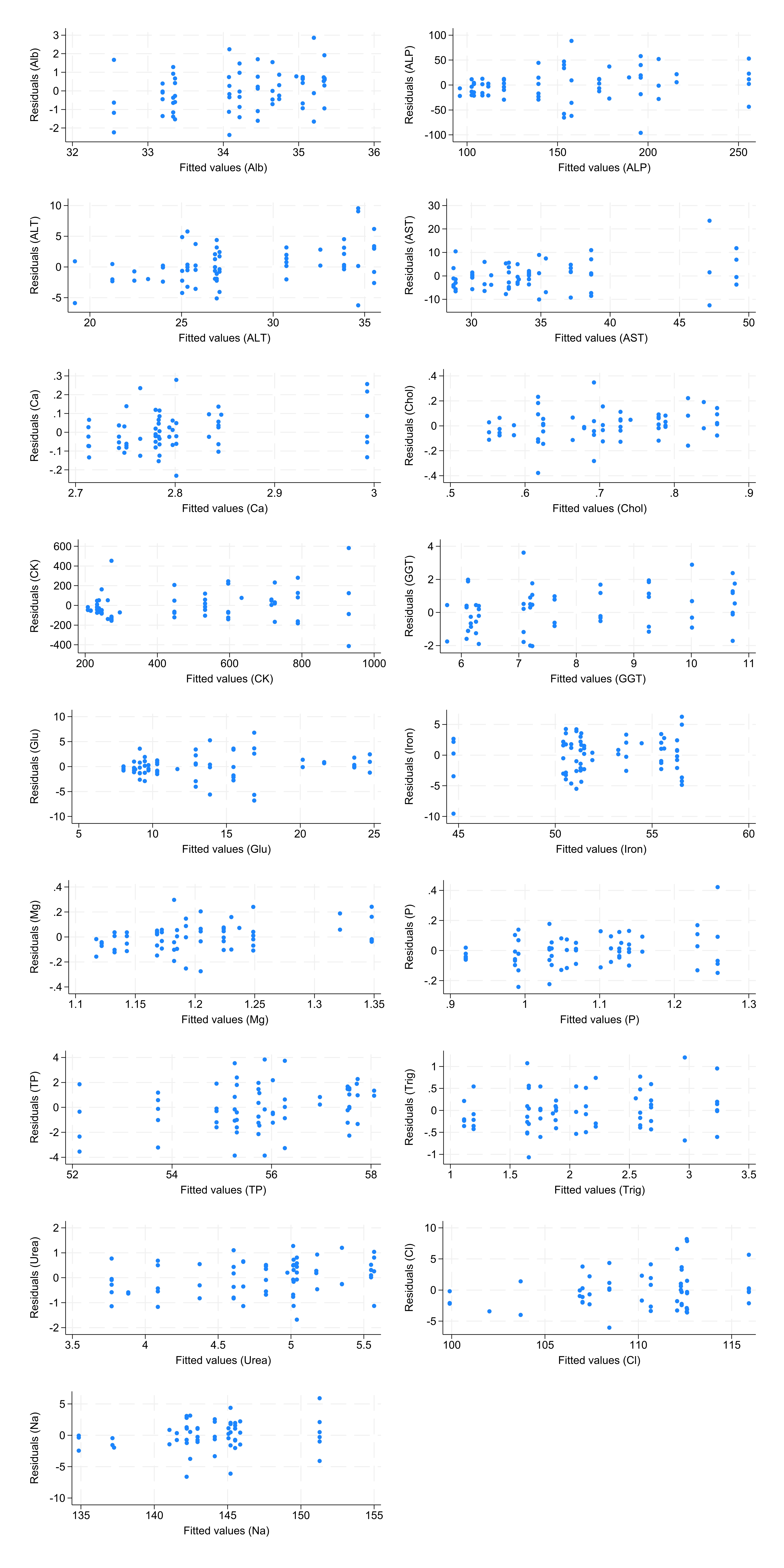

Supplement: Supplementary file 1 [file vetsci-10-00621-s001.zip › Supplementary Figure S1.jpg]

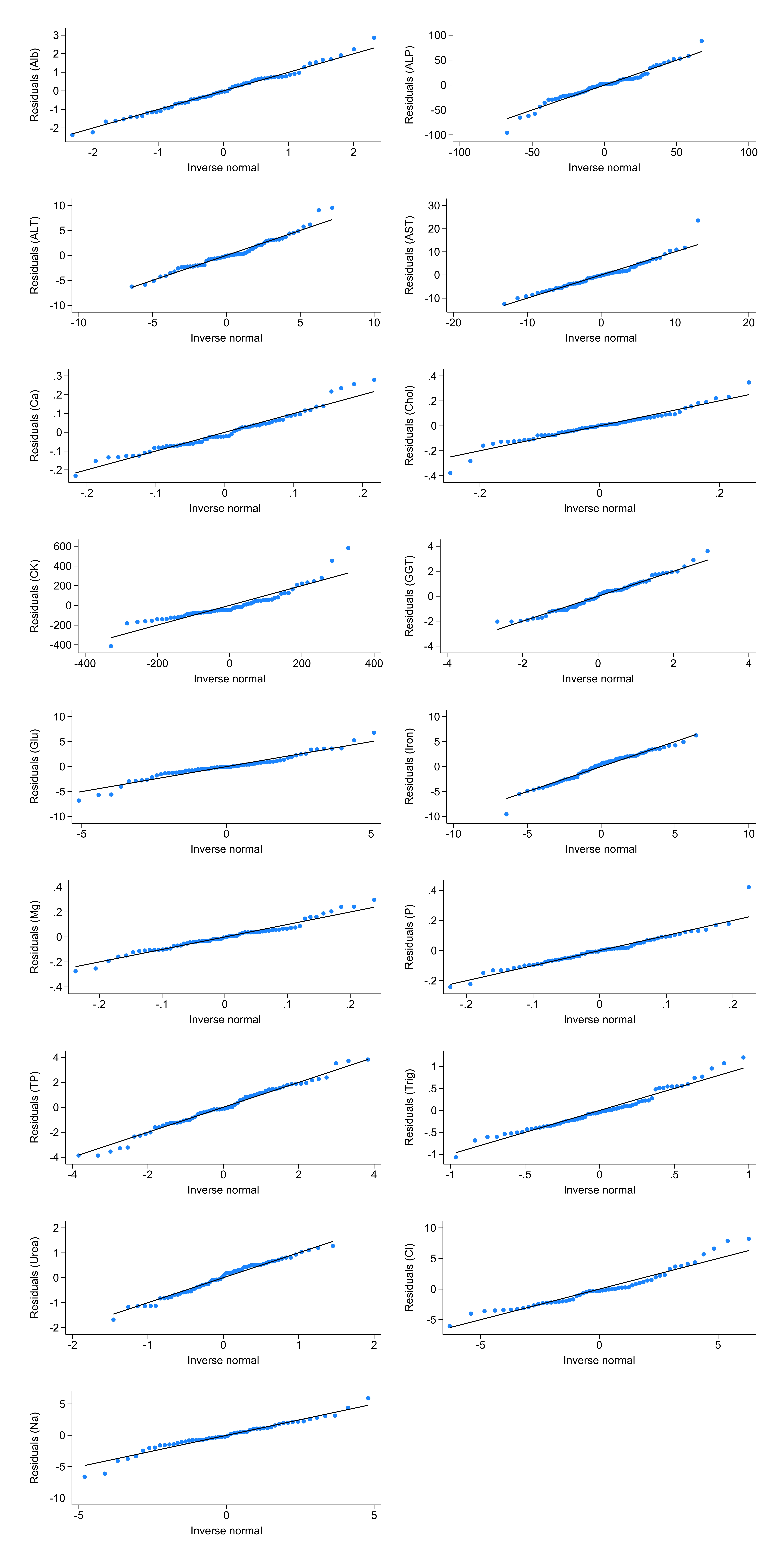

Supplement: Supplementary file 1 [file vetsci-10-00621-s001.zip › Supplementary Figure S2.jpg]

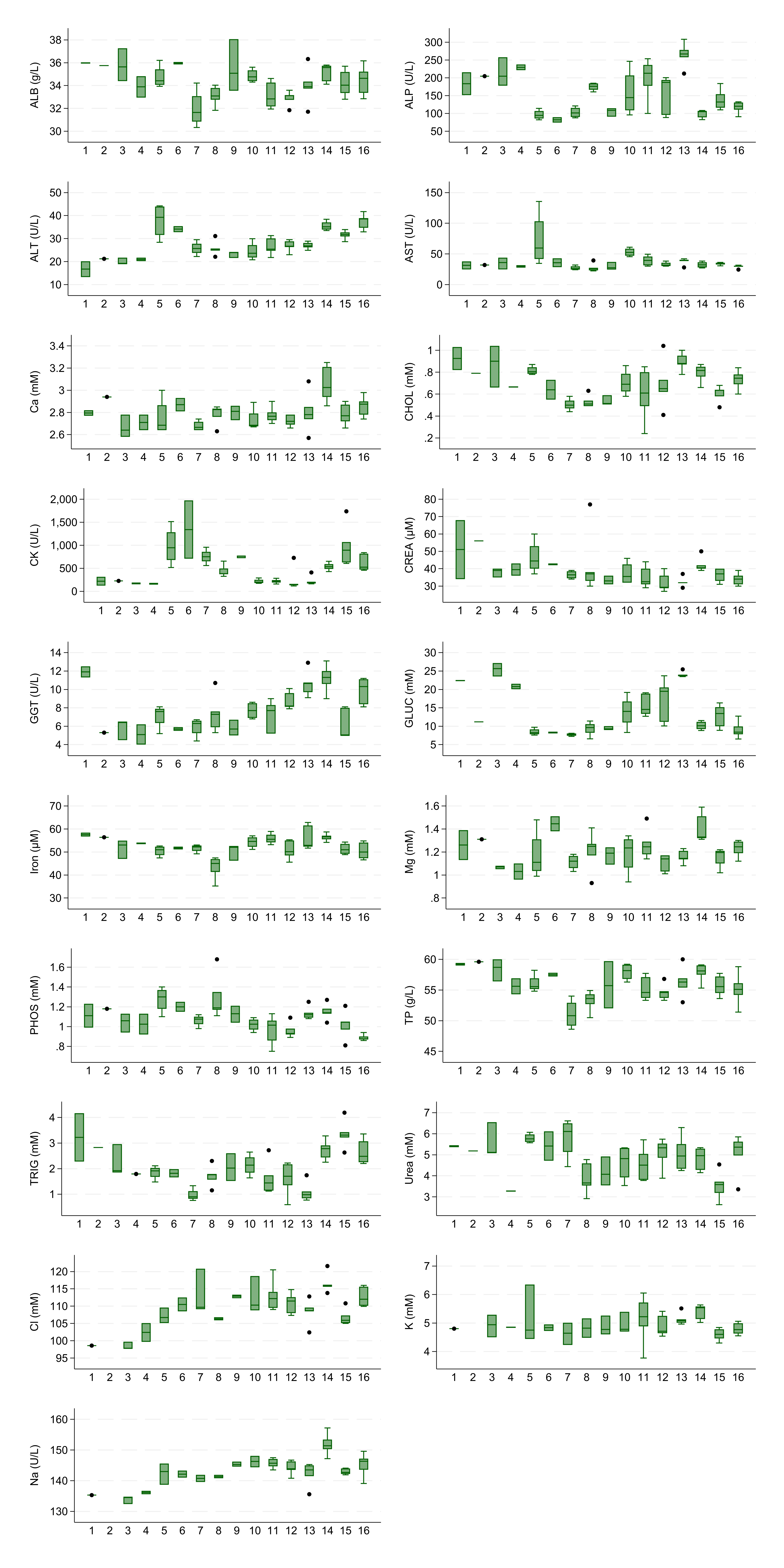

Supplement: Supplementary file 1 [file vetsci-10-00621-s001.zip › Supplementary Figure S3.jpg]
